# Draft Genome Sequences of Isolates from Sediments of the River Elbe That Are Highly Tolerant to Diclofenac

**DOI:** 10.1128/MRA.00849-18

**Published:** 2018-08-16

**Authors:** José Miguel Quesada, Inés Aguilar, Jesús de la Torre, Regina-Michaela Wittich, Pieter van Dillewijn

**Affiliations:** a Department of Environmental Protection, Estación Experimental del Zaidín, Consejo Superior de Investigaciones Científicas, Granada, Spain; University of Maryland School of Medicine

## Abstract

Here, we report the genome sequences of one Achromobacter and four Pseudomonas strains isolated from sediments of the River Elbe which are highly tolerant toward the xenobiotic target compound diclofenac, a nonsteroidal anti-inflammatory drug (NSAID) and emerging contaminant.

## ANNOUNCEMENT

Diclofenac, an over-the-counter medication used in many countries, has a yearly production volume of about 90 tons in Germany and 800 tons in India. Insufficient removal by wastewater treatment plants has led to an increasing occurrence of diclofenac, as well as that of other pharmaceuticals and personal care products (PPCPs), in surface waters such as the River Elbe in Germany (1). Moreover, its toxic effects toward microbes as well as higher organisms (2, 3) have converted diclofenac into an emerging contaminant.

Isolates were obtained from enrichment cultures of sediment samples from the River Elbe downstream of the Hamburg harbor (4) with 0.5 mM diclofenac as the sole carbon and energy source. Bacteria growing in the presence of diclofenac were isolated and identified by sequencing the corresponding 16S rRNA genes. A MIC(s) study with various concentrations of diclofenac in lysogeny broth (LB) medium showed that the isolates RW405 and RW409 grew at concentrations greater than 1,500 mg/liter, but RW407, RW408, and RW410 grew only in a range between 750 and 1,200 mg/liter.

To characterize each isolate, pure cultures of each strain were grown overnight at 30°C in LB medium under agitation. Total genomic DNA was extracted using the Wizard genomic DNA purification kit (Promega). The quality and quantity of the DNA were assessed with the Nanodrop ND-1000 spectrophotometer (Thermo Fisher Scientific). To obtain the draft genome of each isolate, 300-bp paired-end sequencing libraries were prepared using the Illumina Nextera XT DNA library version 3 sample preparation kit, and sequencing was performed with the Illumina MiSeq platform at the Center for Scientific Instrumentation of the University of Granada (Spain). Sequence reads were assessed for quality using FastQC (Babraham Bioinformatics, Q > 30), filtered using Trimmomatic (5), and assembled *de novo* with Velvet (version 1.2.10) and VelvetOptimiser (version 2.2.5) (6) within a customized workflow on Galaxy (http://galaxy-mel.genome.edu.au/galaxy/). The contigs obtained were further annotated with the Rapid Annotations using Subsystems Technology (RAST) server version 2.0 (7), and for submission to GenBank, gene annotation was performed using the NCBI Prokaryotic Genome Annotation Pipeline (PGAP) (8).

Genome sizes vary between 5,862 and 7,346 Mbp and GC contents from 61.8 to 67.6% (Table 1). Taxonomy was determined by comparing 16S rRNA gene sequences in EzBioCloud (9) and the *rpoD*, *recA*, and *gyrB* genes (10) with a BLAST search (11). As possible mechanisms for diclofenac tolerance, the isolates harbored between 35 and 46 genes related to efflux mechanisms for multidrug resistance and between 143 and 240 genes related to the metabolism of aromatic compounds, which constitute between 0.6 and 0.7% and 2.4 and 3.8% of the total number of genes, respectively. Pairwise similarity between the genomes as determined by average nucleotide identity (ANI) with the OrthoANIu tool (12) ranged between the pseudomonad genomes from 76.6 to 82% and between the pseudomonads and the *Achromobacter* strain from 69.2 to 70.7%, which are all well below the 95 to 96% species threshold (13).

**TABLE 1:** Characteristics and accession numbers of genomes of the diclofenac-tolerant bacterial isolates

Isolate	Bacterial species^*a*^	Genome size (bp)	No. of contigs	*N*_50_ (bp)	Total no. of genes	G+C content (%)	No. of genes related to efflux systems^*b*^	No. of genes related to metabolism of aromatic compounds^*b*^	GenBank accession no.
RW405	Pseudomonas putida	5,862,946	33	127,964	5,612	61.8	35	152	QHJD00000000
RW407	Pseudomonas citronellolis	7,346,097	108	38,667	6,724	67.4	37	256	QGSL00000000
RW408	Achromobacter xylosoxidans	6,532,250	49	57,962	6,060	67.6	44	143	QHHO00000000
RW409	Pseudomonas chlororaphis	7,154,805	42	130,324	6,555	62.5	46	171	QHHP00000000
RW410	Pseudomonas aeruginosa	6,529,102	74	120,648	6,289	66.2	42	166	QGSM00000000

a Strain identification by ≥99% similarity with 16S rRNA and ≥97% with the *rpoD*, *recA*, and *gyrB* genes.

b According to annotation with the RAST server.

### Data availability.

The whole-genome sequences have been deposited in DDBJ/ENA/GenBank under the accession numbers listed in Table 1. The versions described in this paper are the first versions.
